# Nanoscale Estimation of Coating Thickness on Substrates via Standardless BSE Detector Calibration

**DOI:** 10.3390/nano10020332

**Published:** 2020-02-15

**Authors:** Radim Skoupy, Tomas Fort, Vladislav Krzyzanek

**Affiliations:** Institute of Scientific Instruments of the Czech Academy of Sciences, Kralovopolska 147, CZ-61264 Brno, Czech Republic; radim.skoupy@isibrno.cz (R.S.); fortt@isibrno.cz (T.F.)

**Keywords:** SEM, quantitative imaging, back-scattered electrons, standardless calibration, electron mirror, sample bias, Monte Carlo simulation, thin coating layers

## Abstract

The thickness of electron transparent samples can be measured in an electron microscope using several imaging techniques like electron energy loss spectroscopy (EELS) or quantitative scanning transmission electron microscopy (STEM). We extrapolate this method for using a back-scattered electron (BSE) detector in the scanning electron microscope (SEM). This brings the opportunity to measure the thickness not just of the electron transparent samples on TEM mesh grids, but, in addition, also the thickness of thin films on substrates. Nevertheless, the geometry of the microscope and the BSE detector poses a problem with precise calibration of the detector. We present a simple method which can be used for such a type of detector calibration that allows absolute (standardless) measurement of thickness, together with a proof of the method on test samples.

## 1. Introduction

A scanning electron microscope (SEM) is a powerful method for examining many types of samples [1,2,3,4]. As the primary electron beam interacts with a sample, a wide spectrum of signals, carrying information about different physical properties of the sample, is generated [5]. The detection possibilities differ among individual types of SEMs, but secondary and back-scattered electron signals are the most common ones. We focus in detail on calibrated image acquisition of back-scattered electrons (BSE) using a retractable circular BSE detector (CBS), which compared to in-lens BSE detectors offers well-defined detection geometry without distortions caused by the optical system of the SEM column (in the field free mode of the final demagnifying lens).

BSE imaging (BSEi) is an often-used method for differentiation of elements with a different atomic mass in a sample thanks to its mass contrast [5]. The signal captured by the BSE detector is dependent on several factors such as elemental composition and its corresponding back-scattering coefficient, local thickness of individual layers or grains, local density, energy of the primary electron beam, collecting angle of the BSE detector and application of beam deceleration [6,7,8].

We have continued previous work where the back-scattering coefficient is estimated as a function of the over-layer thickness [9,10]. The authors there used Pd and Au layers on a Si substrate, the thicknesses of which were estimated using calibrated BSE imaging together with Monte Carlo simulation. Unlike the authors of that study, we focus on much smaller thicknesses (<25 nm). The BSE coefficient η is theoretically described for those thicknesses using an example of an Au layer on a C substrate and a C layer on an Au substrate in [11].

Calibration of the BSE detector response to the primary electron beam plays a crucial role for precise data acquisition and corresponding data processing where captured data are compared with simulated signal intensities. The limits or calibration points bring a connection between the experiment and simulation and thus allow assigning the correct covering layer thickness to the corresponding BSE signal. Several approaches for calibration of the BSE signal have recently been published:

*One tail relative calibration—upper limit* [9] where the BSE images of thin Pd layers on a silicon wafer are normalized by the BSE signal of bulk Pd. The same detector settings for all samples were achieved by simultaneous imaging of all four samples (Pd bulk, Pd 10 nm, Pd 110 nm, and Pd 270 nm), and the BSE intensities are compared to each other. The same calibration was applied also in the case of measurement of Au layers on a Si substrate [10], where the upper limit is a measured BSE coefficient from bulk Au.

*One tail relative calibration—bottom limit* [12] is based on image background measurement in the vacuum part of a “sample”. The upper limit is then floating just under the saturation limit of the detector. This detector calibration was applied for the determination of the In${}_{x}$Ga${}_{1-x}$/GaAs ratio in the wedge-shape sample that was prepared by a gallium focused ion beam SEM (FIB-SEM). The density of a sample with changing composition was determined by linear interpolation of pure GaAs and InAs densities. The local thickness is calculated from the sample geometry of the wedge shape. Such measurements were analogically performed also for very thin samples using a scanning transmission electron microscopy (STEM) detector [13].

*Two tail relative calibration* [14], where the captured signal is normalized between two defined limiting values. Here, the BSE signal of the Si substrate is used as the lower limit and the signal of the Au substrate is used as the upper limit. Another combination of limits is the detector response to the blanked beam as the lower limit and the BSE signal of the crystalline Si sample as the upper limit [15].

*Multi-point relative calibration* [16] uses 26 mineral standards describing the correlation of the BSE signal on the changing atomic number *Z* in the range from 10.41 to 73.16. The atomic number *Z* of an unknown sample can be determined with maintaining the imaging conditions from the measured BSE signal—*Z* dependency.

All of the above-mentioned methods for calibration of the BSE detector are relative to chosen limits and therefore dependent on the calibration sample. The disadvantage is the possible variation of those limits with the use of different calibration standard qualities, cleanliness, and homogeneity.

The principle of quantitative BSE or STEM imaging is based on a comparison of captured and normalized image data with a theoretical model. Although a few empirical equations describing the BSE yield have been described [17], in practice, computer simulations usually based on the Monte Carlo methods are typically used which also include the SEM column and the BSE geometries. There are several types of software available where electron–matter interaction can be simulated. They differ in the incorporated physical models, the possibilities for sample definition, data exporting, or computed physical characteristics. The principle of quantitative STEM imaging is described in more detail in [18,19,20].

**Figure 1:**
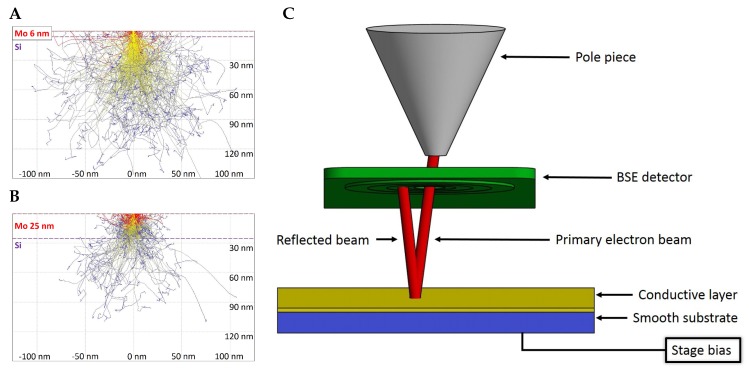
(**A**,**B**) trace of primary electrons hitting the sample, showing the principle of signal change accordingly to increasing coating layer thickness. The back-scattering coefficient is increasing with thickness of the coating layer (in the case that the covering layer has higher back-scatter electron (BSE) yield than the substrate). For example, in the case of a 6 nm thick layer of Mo on a Si substrate (**A**) most of the primary electrons penetrate the Mo layer and thanks to the lower BSE yield of Si the resulting BSE coefficient is lower than in the case of 25 nm Mo (**B**) where nearly all electrons are absorbed or back-scattered in the layer, simulated in CASINO version 2.51; (**C**) principle of the calibration of full beam on the BSE detector. Primary electron beam (red) is reflected on biased conducting surface (gold) created on smooth substrate (blue) back to the BSE detector (green).

The maximum measurable layer thickness by qBSEi is given by the acceleration voltage and the corresponding penetrability into the sample [21]. Higher acceleration voltage brings a wider measurable range of thicknesses, but with lower modulation of the signal by unit thickness change. It follows that, for high-precision with thin layers, a lower acceleration voltage is preferred and, for a high measurable range of thicknesses, a higher beam energy should be used. An example of the penetrability of a 3 kV electron beam is shown in Figure 1A,B, where the beam partially goes through 6 nm of Mo but is fully absorbed in a layer of 25 nm.

## 2. Materials and Methods

### 2.1. Microscope Used

The test samples were imaged in the field free mode of a scanning electron microscope Magellan 400 L (FEI-Thermo Fisher Scientific, Waltham, MA, USA) equipped with a commercial four annular segment CBS detector (FEI). The imaging was performed with the following settings: the acceleration voltage of 3 kV, magnification in the range from 1000 to 500,000×, the probe current of 50 pA, resolution of 1024 × 884 pixels in each individual recorded image, the working distance of 4 mm, the distance from the pole-piece to the active detector plane of 1 mm, and the dwell time of 10 μs. All images were captured as 16 bit to keep high dynamics. Well-adjusted SEM including a centered BSE detector according to the optical axis is a prerequisite for quantitative measurements. For data collection, the innermost detector segment of the BSE detector (S1) was used because it is possible to take the calibration image of the whole segment under the same imaging conditions. The active area of the annular segment S1 is given by the diameter range from 0.75 to 1.25 mm that, for the above-mentioned settings, corresponds to the collection angles in the range of 124.4 to 205.4 mrad.

### 2.2. Absolute Detector Calibration

We present a two-tail absolute calibration method for a BSE detector that uses the detector response to no impacting electrons as a bottom limit and all primary electrons impacting the detector for the upper limit. Moreover, the method includes correlation of the captured BSE signal with the position and energy of the impacting electrons.

The background level of the BSE detector that is related to the brightness setting of the SEM is determined when no beam is hitting the sample, i.e., the lower calibration limit is calculated as the mean value of all pixel values in the recorded beam blanked image. It is necessary to avoid under-saturation. The acquired images are then normalized between those limiting values by the equation:

$$I_{norm}=\frac{I_{sample}-I_{blank}}{I_{full}-I_{blank}}$$

where $I_{norm}$ is the normalized image intensity towards the actual beam current and detector contrast/brightness settings, $I_{sample}$ is the original intensity, $I_{blank}$ is the mean value in blanked image, and $I_{full}$ is the signal intensity of full beam on the detector.

In the case of calibration of the BSE detector using the full beam, the geometry of the detector inside the microscope chamber does not allow for performing this directly as is typically used in qSTEM. The STEM detector is located under a sample and primary electrons may impact the STEM detector directly, in the case of the retractable BSE detector that is located immediately under the SEM column pole piece and therefore not in the field of view for the primary electrons. However, the solution could be to reverse the electron trajectories by applying appropriate negative stage bias to the sample and impact of the electrons on the BSE detector from its sensitive side as shown schematically in Figure 1C. For that purpose, a gold coated circular piece of glass with a diameter of 10 mm was prepared. Note that it is necessary to avoid over-saturation.

Unfortunately, in the case of the microscope used, the primary beam energy set by the user is the landing energy and so the SEM operating software raises the primary beam energy when negative stage bias is applied. In order to overcome this limitation, an external voltage source BERTAN 225 (Spellman, New York, NY, USA) connected directly to the sample stage was used.

**Figure 2:**
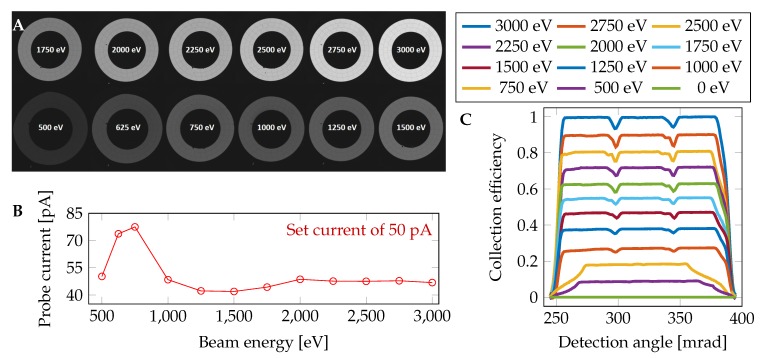
(**A**) set of BSE detector calibration images for the innermost segment at various electron energies of the primary beam. The change in diameter of the detector segment at individual electron energies is caused by image distortions during its reflection; (**B**) probe current measurements at used electron energies. Together with changing the electron beam energy, the probe current impacting the sample is changing and it is necessary to correct it; (**C**) calibration of the BSE detector according to primary beam energy and position of electron impact. Because of the rotational symmetry of the detector, only the back-scattering angle is considered. A working distance of 4 mm and a distance of the pole piece and the detector of 1 mm were used.

For precise simulation of the captured signal, the detector response factor (DRF) for the electrons with different energies and positions of impact is introduced as follows. In order to estimate the DRF, a series of BSE detector images (Figure 2A) with a beam energy in the range from 500 to 3000 eV was captured. In the captured images of the BSE detector, the centers of the individual segments were detected and circular averages were performed (shown in Figure 2C). The results were used (after interpolating to non-measured energies) to determine the space and energy-dependent DRF for each individual simulated back-scattered electron. The actual beam current (Figure 2B) was measured by the Faraday cup mounted on the microscope stage using the picoammeter KEITHLEY 6485 (Keithley Instruments, Cleveland, OH, USA).

### 2.3. Test Samples

Three types of representative samples were used to show the proof of the described method. Very thin layers of chromium, molybdenum, and gold of known thickness were sputtered on a silicon wafer. The accurate thicknesses of the sputtered thin layers were estimated by the calibrated sputtering speed and the number of passes under the sputtering head. The sputtering speed was estimated from the thickness of the layer estimated by profilometry and the known number of passes. A cleaned silicon wafer with size of 10 × 10 mm was used as the substrate in all cases.

The chromium and molybdenum layers were prepared in an sputtering system B-301-308 (AURION, Seligenstadt, Germany) at a pressure of $9.9\times 10^{-5}$ Pa. The target was cleaned in an Ar athmosphere for 300 s by discharge. The high-frequency source power was 300 W. The flow of Ar was 20 sccm. The substrates were cleaned before covering by glow discharge with a power of 150 W for 120 s (rotation regime with speed 9 rpm, Ar pressure of 0.274 Pa).

In the case of molybdenum, the voltage on the magnetron was stabilized to −62 V. The pressure of the working gas in the chamber during sputtering was 0.162 Pa. The deposition was done in pulse regime $\pm 45^{\circ }$ and at a speed of 1.536 rpm. The thickness after 180 s sputtering was estimated by a profilometer Taylor–Hobson Talystep as 30 nm. For a layer thickness of 25 nm, the time was reduced to 150 s, for 20 nm to 120 s, for 15 nm to 90 s, for 8 nm to 48 s, for 3 nm to 18 s, and for 1 nm to 6 s.

In the case of chromium, the voltage on the magnetron was −52 V, and a chamber pressure of 0.160 Pa, a speed of 1.536 rpm, and a sputtering time of 400 s were used with a resulting layer thickness of 92 nm. The deposition time for the 25 nm layer was 108 s, for 20 nm 87 s, for 15 nm 65 s, for 8 nm 35 s, and for a 3 nm layer 13 s and 5 s for 1 nm.

The gold layers were prepared at room temperature from the gold target of (99.9% purity) in an argon (99.999%) discharge in a commercially available Z 550 RF magnetron sputtering plant (Leybold Heraeus, Cologne, Germany). During the deposition, the power was held at 100 W and the pressure of argon was 0.150 Pa. The evacuation system consists of a rotary and turbomolecular pump, and limited pressure is 10${}^{-4}$ Pa. In such a way, a series of layers with thicknesses of 1.7, 3.4, 5.1, 6.7, 8.5, 11.9 and 13.4 nm were prepared.

### 2.4. Theoretic Description of the BSE Signal

The signal captured by the BSE detector is given by the back-scattering coefficient, angular distribution of BSEs, energy of individual BSEs, and local sensitivity of the BSE detector used (discussed in more detail in Section 2.2). In this part, we focus on the simulation of electron–matter interaction and the calculation of the amount of energy transferred to BSE detector from a sample with a known composition and detection geometry.

The Monte Carlo simulations, which describe well the electron scattering in a sample of matter, were done in the software CASINO version 3.3.0.4 [22] in an appropriate range of coating layer thicknesses from 0 to 28 nm (Cr and Mo) and from 0 to 16 nm for Au. Several physical models for total and partial cross sections such as Mott, Rutherford, and Reimer models were investigated, but, finally, the physical model Elsepa [23] was used in this work as it gives the most accurate results.

**Figure 3:**
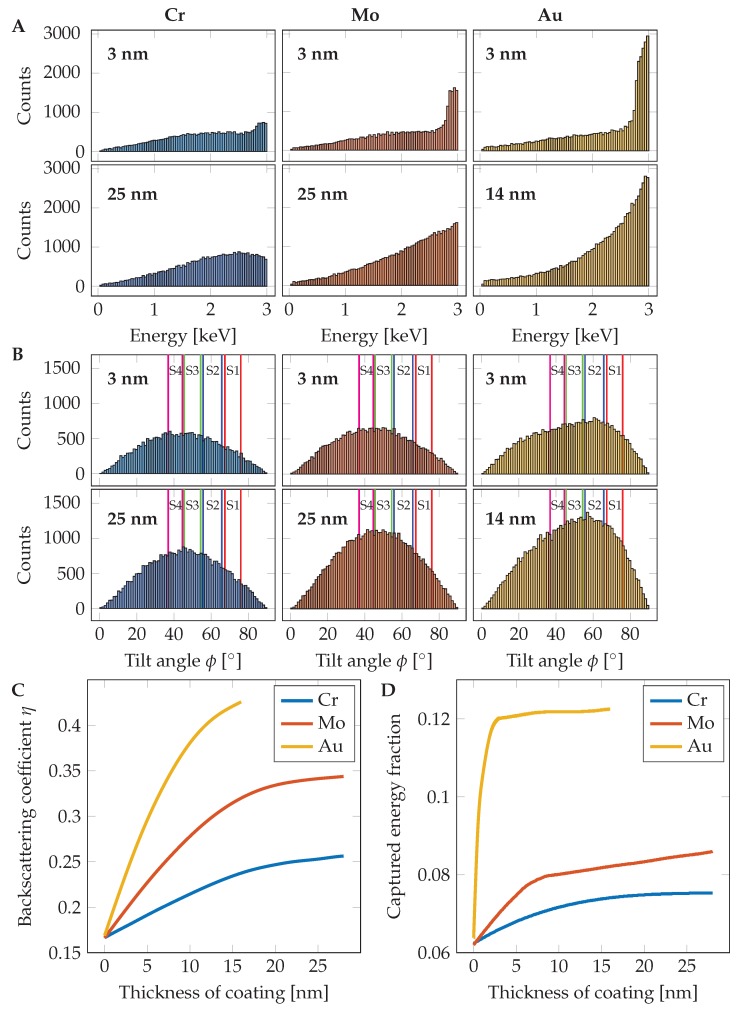
Monte Carlo simulation based inputs of the method. (**A**) With increasing coating thickness, the amount of middle energy BSE increases and enhances the total energy emitted from the sample. The peak just under the landing energy of 3 kV is caused by reflection in the coating layer. The rest of the energy spectrum is caused by the Si substrate. (**B**) The elements with higher atomic number reflect the impacted electrons closer to the optical axis (ϕ=0 is plain of a sample surface) and thus increase the signal captured by the BSE detector. Collecting angles of individual detector segments S1–S4 is highlighted; (**C**) dependency of back-scattering coefficient η on the thickness of Cr, Mo and Au coatings on a Si substrate; (**D**) captured energy fraction according to the chosen element and coating thickness. The fraction was calculated from the scattering angle and energy of each BSE.

Each simulation was based on 300,000 impinging electrons in order to suppress statistical error. Simulated BSE coefficients accordingly to the coating thickness are shown in Figure 3C and the calculated captured energy fraction (CEF) in Figure 3D. The fraction of detected energy is estimated from information about energy and back-scattering angle (an example for 3 nm and 25 nm layers can be seen in Figure 3A,B) of each simulated electron as used in [24,25]. For data processing, the results of the MC simulation were analyzed in MATLAB (Mathworks).

The simulated BSE signal $SBS_{x}$ generated by coating layer of element α with thickness *x* on the substrate β is calculated by the equation:

$$SBS_{x}=\eta_{x}\cdot CEF_{x}=\eta_{x}\cdot \frac{\sum_{i=1}^{c}E_{i}\cdot DRF_{E,\sigma }}{\sum_{i=1}^{t}E_{i}\cdot DRF_{E}},$$

where $\eta_{x}$ is the BSE coefficient of a layer with thickness *x*, $E_{i}$ is the energy of the electron *i*, *c* is the amount of the BSE captured by the detector, *t* is amount of all BSE and $DRF_{E,\sigma }$ is the detector response factor of an electron with energy *E* and position of impact given by the angle σ. The atomic number $Z_{\alpha }$ is higher than $Z_{\beta }$ in the case of test samples.

## 3. Results

**Figure 4:**
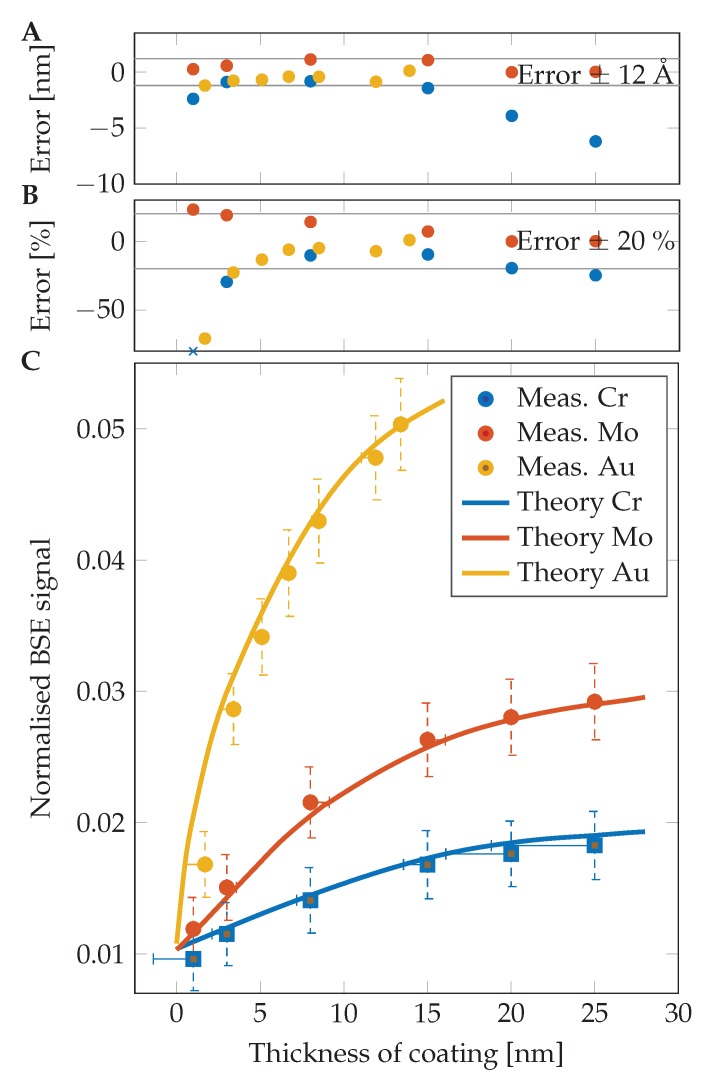
Results of qBSE imaging. (**A**) comparison of measured and nominal thicknesses. Most of the measurements show an error lower than 1.2 nm; (**B**) comparison of measured and nominal thicknesses. Most of the measurements show an error lower than 20%. The blue × mark shows a data point out of the used *y*-axis range; (**C**) theoretical BSE signal captured by an S1 segment in WD of 4 mm for Cr, Mo, and Au. The individual points show mean values of measured samples with its standard deviation and horizontal lines indicate the thickness assigned by qBSEi. The oxidized layer of Cr is highlighted by square marks.

Thicknesses of coating layers on prepared samples with very good precision were measured using the herein-described new method. A higher error is visible in the case of a low-thickness region, where the simulation does not match experimental measurements very well. As visible in the graphs in Figure 4 and numerically in Table 1, the results for chromium are lower than expected in all examined cases. This could be caused by oxidation of those layers during the transfer of the sample from the sputter coater chamber to the SEM in air. An additional experiment had proven the oxidation of the chromium layers and consequently the change in estimated thickness (MC simulations are done for pure Cr). For suppression of such surface change caused by air exposure, a sample of chromium was prepared in the sputter coater ACE600 (Leica Microsystems, Vienna, Austria) and directly transferred into the SEM chamber via a vacuum transfer shuttle VCT100 (Leica Microsystems, Vienna, Austria) without leaving the high vacuum. The thickness of the layer was estimated as 11.14 nm in the case of no air exposure and 9.37 nm after 30 min in air. The results after one more hour in the air were the same as after half an hour. This shows fast oxidation of the pure chromium surface and creation of a Cr${}_{x}$O${}_{y}$ layer.

In the case of Mo and Au layers, the estimation error is decreasing with the increasing thickness of the coating layer. In the case of Mo, the error is decreasing from 23% (1 nm) through 7% (15 nm) to 0.1% for a 25 nm thick layer. The Au gives a high error of 70% at 1.7 nm, but the error is rapidly decreasing up to an accuracy of 0.9% in the case of a 13.4 nm layer. All results are shown in Table 1.

**Figure 5:**
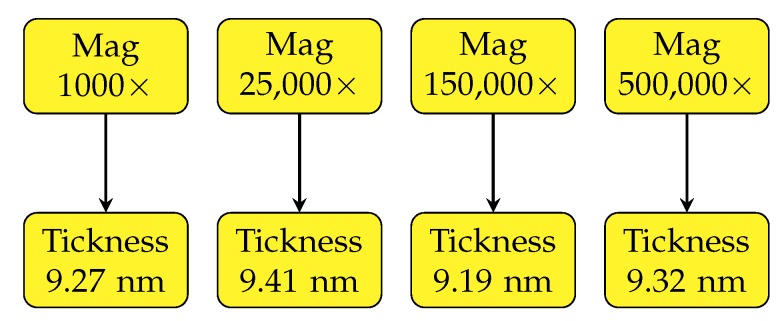
Change of estimated thickness of a molybdenum layer on a silicon wafer according to image magnification.

An advantage of the presented method is a wide magnification range and the corresponding spatial resolution which can be achieved. In general, it is based only on maximum resolution of the SEM used and the rapidity by which a creation of hydrocarbon contamination layer is created which decreases the measured thickness. For estimation of the change of the estimated coating layer thickness, the molybdenum sample with a nominal thickness of 8 nm was used (the thickness estimated by qBSEi is around 9.3 nm). A comparison of measurements at a wide range of magnifications from 1000× up to 500,000× is shown in Figure 5. We found high accuracy across all the magnification range with a deviation around 0.1 nm. This brings an opportunity to measure the space dependency of coating thickness at all magnifications.

## 4. Discussion and Conclusions

We presented and proved a new calibration method for retractable BSE detectors mounted in the vacuum chamber of an SEM. Using the electron mirror BSE detector calibration, it is possible to measure the response of the detector to the primary beam, which is necessary for precise measurements and comparison of measured images. Using this approach, it is possible to measure the energy and spatial dependent response of the BSE detector which is usually unavailable without detector dismounting.

The measured thicknesses match the nominal ones very well (note that the nominal thicknesses are not entirely accurate and may slightly vary) because we achieved an accuracy of around several tenths of a nm in most cases. Thanks to the precise BSE detector calibration, we achieved better results with low-thickness films than is described in [9], where the thickness of a 10 nm Pd layer was immeasurable and, in the case of a 25 nm Au layer, an error of 20% was attained [10]. We achieved an error lower than 10% for an Au layer down to a thickness of 6.7 nm and, even in the case of a 5.1 nm Au layer, the error was only 13%. The high error at lower thicknesses (3.4 and 1.7 nm) is probably caused by low layer thickness homogeneity and creation of “metal islands” on the substrate during preparation of the layer as shown in [26]. Another source of inaccuracy is the sample being covered by a hydrocarbon contamination layer, which changes the BSE coefficient and the BSE energy distribution accordingly to its thickness and corresponding penetrability of the primary electron beam through the contamination layer [6,11,27,28].

For its accuracy, the method can be used as a non-destructive control of thin layer thickness during the preparation process in the semiconductor industry or manufacturing of thin coatings on optical elements.

Further improvement can be achieved by using all available BSE detector segments for image acquisition. This upgrade requires complex calibration of each individual segment and measuring its angle/space-dependent response factor. We would like to focus on this in a subsequent study. We suppose that a measurement accuracy around 0.5 nm is possible with enhanced detection angles, a higher number of simulated electrons, and precise positioning of the BSE detector in the SEM chamber. 

## Figures and Tables

**Table 1:** Results of qBSEi measurements.

	Nominal	qBSEi	Error	Error
	[nm]	[nm]	[nm]	[%]
Au	1.7	0.50	−1.20	−70.8
	3.4	2.63	−0.77	−22.7
	5.1	4.42	−0.68	−13.3
	6.7	6.29	−0.41	−6.1
	8.5	8.08	−0.42	−4.9
	11.9	11.04	−0.86	−7.2
	13.4	13.52	0.12	0.9
Mo	1.0	1.23	0.23	23.1
	3.0	3.57	0.57	19.0
	8.0	9.13	1.13	14.1
	15.0	16.07	1.07	7.1
	20.0	19.99	−0.11	−0.1
	25.0	25.03	0.03	0.1
Cr	1.0	−1.39	−2.39	−239.4
	3.0	2.15	−0.89	−29.5
	8.0	7.18	−0.82	−10.3
	15.0	13.56	−1.44	−9.6
	20.0	16.09	−3.91	−19.5
	25.0	18.81	−6.19	−24.8

## Abbreviations

The following abbreviations are used in this manuscript:

BSE	Backscatter electron
BSEi	Backscatter electron imaging
CBS	Circular backscatter detector
CEF	Captured energy fraction
DRF	Detector response factor
EELS	Electron energy loss spectroscopy
MC	Monte Carlo
qBSEi	Quantitative backscatter electron imaging
qSTEM	Quantitative scanning transmission electron microscopy
SBS	Simulated backscattered electron signal
sccm	Standard cubic centimeters per minute
SEM	Scanning electron microscopy
